# Supplementary material for: Complementary Therapy Learning in the Setting of Lung Transplantation: A Single-Center Observational Study of Appropriation and Efficacy
Source: J Clin Med. 2023 Feb 21;12(5):1722. doi: 10.3390/jcm12051722 (PMC10002550; doi:10.3390/jcm12051722)
Supplement: Supplementary file 1 [file jcm-12-01722-s001.zip › Supplementary File S1 (Study Protocol - Techniques)vuPG.pdf]

Supplementary File S1 (Study Protocol - Techniques)

Relaxation was taught with the help of a smartphone application (RespiRelax). During the training session the patients were asked to breathe regularly for five minutes while silently counting (three seconds inhaling and three seconds exhaling), following a bubble on the smartphone moving up and down. While they were following the bubble, they were asked to relax the shoulders, then the jaws and then all the body through a body scan. Patients were asked to practice this exercise three times a day, for five minutes. They were told that the aim of this exercise was to increase their muscular relaxation, to decrease stress, to decrease the sympathetic tonus, and to favor the parasympathetic state. They could also use it to focus their attention during an uncomfortable procedure. The technique was taught by two psychologists and two anesthesiologists in the same way.

The self-hypnosis group was taught during a 20-minute session. The same type of induction and therapeutic suggestions was used by the investigators without a script. Induction was based on regular respiration, focus on surrounding sounds, specific areas of the body, and therapeutic suggestions were based on the patient's own memories. The patients were taught to visualize themselves in a safe place, or doing their favorite activity, they could learn how to increase their comfort and to "protect" themselves in an uncomfortable situation. For example, the metaphor of a magic glove protecting the hand and the wrist was taught to increase the comfort during blood gas sampling. At the end of the session, patients received a CD or a link (https://www.dropbox.com/s/ax2hsupg8e56bnf/fauteuil%20de%20nuages.wav?dl=0) with relaxation and self-hypnosis exercises (SACEM 215517/2013; https://societe.sacem.fr/en). The patients were told to practice regularly at home, they would use the technique to increase their comfort during an uncomfortable or painful procedure or during a stressful situation (i.e., endoscopic examinations, uncomfortable care, sleep difficulties…). The technique was taught by two psychologists and two anesthesiologists. All of them had done the same academic 1-year hypnosis course at the Paris-Saclay University. They all had more than 8 years' experience at the time of the study. To avoid any discrepancy, they ascertained regularly that they had the same practice.

The sophrology exercise was taught during a 30-minute session with practical physical and mental exercises, using techniques such as concentration, deep breathing, relaxation, visualization, and simple movements. The patients were told to practice regularly at home and to use the techniques as necessary to increase their comfort during an uncomfortable or painful procedure or a stressful situation. The technique was taught by a registered nurse who had 4 years' training and had a master's degree in "sophrologie caycedienne" (https://sofrocay.com/). She recorded the session on the cell phone of the patient for ulterior training.

The holistic gymnastic session consists of learning three short exercises of simple corporal movements, stretching and abdominal breathing. The technique was taught by a physiotherapist who had done a 2-year course in holistic gymnastics. Patients were told to practice these exercises as routine training every day.
