# Supplementary material for: Complementary Therapy Learning in the Setting of Lung Transplantation: A Single-Center Observational Study of Appropriation and Efficacy
Source: J Clin Med. 2023 Feb 21;12(5):1722. doi: 10.3390/jcm12051722 (PMC10002550; doi:10.3390/jcm12051722)
Supplement: Supplementary file 1 [file jcm-12-01722-s001.zip › Supplementary File S2 ( Figure S1 - Correlations).pptx]

## Slide 1
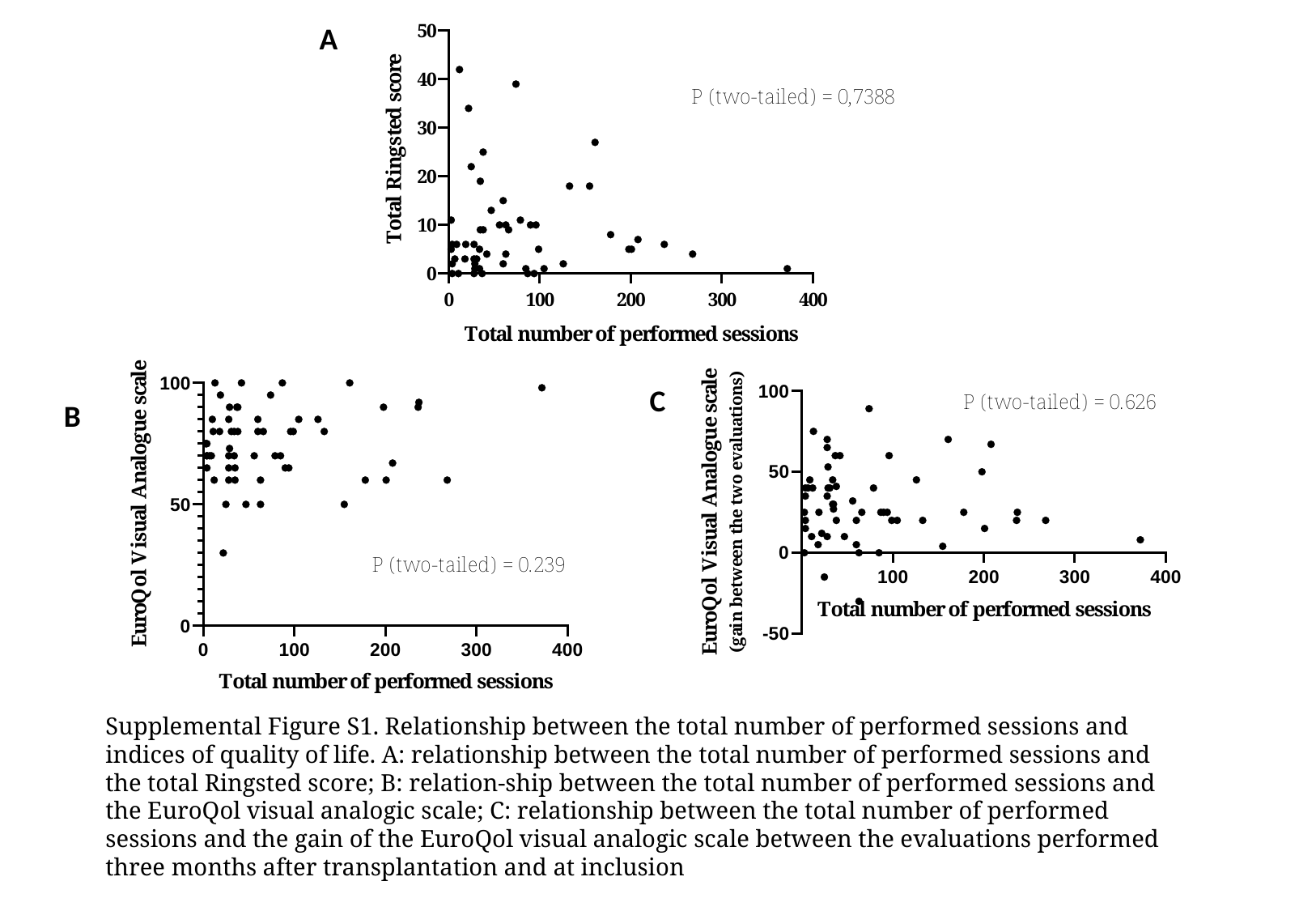

A
C
B
Supplemental Figure S1. Relationship between the total number of performed sessions and indices of quality of life. A: relationship between the total number of performed sessions and the total Ringsted score; B: relation-ship between the total number of performed sessions and the EuroQol visual analogic scale; C: relationship between the total number of performed sessions and the gain of the EuroQol visual analogic scale between the evaluations performed three months after transplantation and at inclusion
